# Consumer concerns: motivating to action.

**DOI:** 10.3201/eid0304.970415

**Published:** 1997

**Authors:** C M Bruhn

**Affiliations:** Center for Consumer Research, University of California, Davis 95616-8598, USA. cmbruhn@udavis.edu

## Abstract

Microbiologic safety is consumers' most frequently volunteered food safety concern. An increase in the level of concern in recent years suggests that consumers are more receptive to educational information. However, changing lifestyles have lessened the awareness of foodborne illness, especially among younger consumers. Failure to fully recognize the symptoms or sources of foodborne disease prevents consumers from taking corrective action. Consumer education messages should include the ubiquity of microorganisms, a comprehensive description of foodborne illnesses, and prevention strategies. Product labels should contain food-handling information and warnings for special populations, and foods processed by newer safety-enhancing technologies should be more widely available. Knowledge of the consequences of unsafe practices can enhance motivation and adherence to safety guidelines. When consumers mishandle food during preparation, the health community, food industry, regulators, and the media are ultimately responsible. Whether inappropriate temperature control, poor hygiene, or another factor, the error occurs because consumers have not been informed about how to handle food and protect themselves. The food safety message has not been delivered effectively.


### Consumer Concerns: Motivating to Action

**Christine M. Bruhn**
University of California, Davis, California, USA

Microbiologic safety is consumers' most frequently volunteered food safety concern. An increase in the level of concern in recent years suggests that consumers are more receptive to educational information. However, changing lifestyles have lessened the awareness of foodborne illness, especially among younger consumers. Failure to fully recognize the symptoms or sources of foodborne disease prevents consumers from taking corrective action. Consumer education messages should include the ubiquity of microorganisms, a comprehensive description of foodborne illnesses, and prevention strategies. Product labels should contain food-handling information and warnings for special populations, and foods processed by newer safety-enhancing technologies should be more widely available. Knowledge of the consequences of unsafe practices can enhance motivation and adherence to safety guidelines. When consumers mishandle food during preparation, the health community, food industry, regulators, and the media are ultimately responsible. Whether inappropriate temperature control, poor hygiene, or another factor, the error occurs because consumers have not been informed about how to handle food and protect themselves. The food safety message has not been delivered effectively.

### Consumer Knowledge and Concern

Consumers are receptive to information about microbiologic hazards. Nationwide surveys by the Food Marketing Institute indicate that more people volunteer concerns about microbiologic hazards than about any other potential food safety issue. From 1992 to 1996, volunteered concern about microbiologic safety increased from 36% to 49% (1). Specifically, concern about contamination by bacteria or other microorganisms was 77%, more than concern about pesticide residues (66%), product tampering (66%), antibiotic residues (42%), or any other food safety risk.

### Food-Handling Practices

Although many consumers recognize the potential seriousness of foodborne bacteria, they lack information on safe handling and storage of food products (2). Williamson et al. (3) found that consumers under 35 years of age knew less about food safety terms and concepts than those over 35. Specific safe food handling was not practiced by 15% to 30% of survey respondents. For example, consumers did not cool cooked food rapidly, with 29% indicating they would let roasted chicken cool completely before refrigerating. Only 32% indicated they would use small, shallow containers to refrigerate leftovers. Consumers did not know that failure to refrigerate may jeopardize safety, with 18% not concerned or uncertain about the safety of cooked meat and 14% not concerned about poultry left unrefrigerated for more than 4 hours. The need for sanitation was not recognized, with only 54% indicating they would wash a cutting board with soap and water between cutting raw meat and chopping vegetables.

Food safety experts have identified the most common food-handling mistakes made by consumers at home. These mistakes include serving contaminated raw food, cooking or heating food inadequately, obtaining food from unsafe sources, cooling food inadequately, allowing 12 hours or more between preparation and eating, and having a colonized person handle implicated food or practice poor hygiene (4). The same factors were identified in mishandling associated with specific pathogens (5).

Address for correspondence: Christine M. Bruhn, Consumer Behavior, University of California, Center for Consumer Research, University of California, Davis, CA 95616-8598, USA; fax: 916-752-3975; e-mail: cmbruhn@udavis.edu.

### Changing Lifestyles

Many factors have contributed to consumers' lack of familiarity with safe food handling and increased foodborne illnesses. Increased participation in the paid labor force has lessened the exposure of young people to food-handling practices in the home; few schools offer or require food preparation classes; and partially prepared foods may have different, less familiar handling requirements (2,6).

People eat out more frequently today, thereby increasing their exposure to the food service industry, noted for high turnover rates and minimal job training in personal hygiene (7). Furthermore, the population is shifting, with an increased percentage of persons at higher risk for foodborne illness because of age or health status (8,9). Additionally, some food safety recommendations related to temperature and acidity do not eliminate risks from some pathogens (2).

### Nature and Source of Foodborne Illness

Consumer perceptions and behavior related to foodborne illness changed little between 1988 and 1993 (10). Consumers misperceived the nature of foodborne illness and the most likely pathogen source. Consumer belief about the type of food responsible for foodborne illness—meat, poultry, seafood, eggs—was consistent with expert opinions; however, consumers believed that foodborne illness was generally mild, without fever, and occurred within a day of eating contaminated food. Infections caused by Salmonella and Campylobacter, the most common foodborne illnesses in the United States (11), are not consistent with the symptoms consumers described, because these organisms have longer latency and cause fever.

Most consumers believed that their foodborne illness was caused by food prepared somewhere other than the home. Williamson (3) found that about one-third of consumers thought food safety problems most likely occurred at food manufacturing facilities, and one-third blamed unsafe restaurant practices. Only 16% thought mishandling was most likely to occur in the home. Fein et al. (10) found that 65% of consumers attributed foodborne illness to food prepared at a restaurant, 17% to mishandling at the supermarket, and 17% to mishandling at home. In contrast, food safety experts believe sporadic cases and small outbreaks in the home are far more common than recognized outbreaks (2).

Failure to recognize the home as a likely source of foodborne illness is not unexpected because illness traced to a food establishment affects many people and may receive widespread publicity (12). Illness that occurs at home is rarely reported unless severe (2).

If consumers misperceive the nature and origin of foodborne illness, they underestimate the frequency of serious consequences and are less motivated to change. Schafer et al. (13) found that motivation for proper food handling requires viewing the mishandling of food as a direct threat to one's health. The failure to associate mishandling of food in the home with foodborne illness interferes with foodborne disease education efforts (10).

### Ubiquity of Organisms

Consumers do not seem to be aware of the ubiquity of microorganisms in the environment. During foodborne disease outbreaks, press accounts focus on fecal contamination of food. Government standards classify natural microorganisms as contaminants, which suggests that microorganisms are only present as a result of mishandling. In contrast, Hazard Analysis and Critical Control Points (HACCP) programs recognize and attempt to control potential dangers related to pathogenic microorganisms.

When consumers in a national sampling were asked on whom they rely for product safety, the percentage responding "myself as an individual" decreased from 48% in 1989 to 25% in 1996 (1). As self-reliance decreased, consumer reliance on food manufacturers and supermarkets increased. This may be a response to the message that if raw food contains microorganisms, it is contaminated. It suggests some consumers are shifting the responsibility for safe food to manufacturers and retailers.

Consumers may not realize they can introduce pathogens during food handling. In 1990, the Food Marketing Institute asked consumers what steps they took at home to ensure the safety of food (14). Respondents volunteered refrigeration (58%), proper storage (35%), checking expiration dates (26%), washing and cleaning the food (25%), cooking properly (22%), and wrapping food properly (20%). No one volunteered washing hands or preventing cross-contamination.

### Labeling

Products must contain safety labels instructing consumers how to handle food. In 1989, the National Advisory Committee on Microbiological Criteria for Foods recommended a mandatory uniform logo for perishable refrigerated foods, uniform labeling for frozen food, "Use by" dates, and time/temperature indicators wherever possible (15).

Although products are currently labeled when they require refrigeration, the label is ineffective because the warning is difficult to find or read. As the proportion of older people increases, print must be larger. Labels should also display symbols to further enhance the effectiveness of the message.

Safe-handling labels on meat products appear to have made a difference. The Food Marketing Institute (1) found that 60% of survey respondents had seen the labels. Of those aware of the labels, 65% said the labels increased their awareness of safety, and 43% said they changed their behavior as a result of the information. The most common volunteered change was washing the counter and utensils after contact with meat (approximately 40%), followed by washing hands more frequently (approximately 20%) and cooking to the proper temperature (approximately 20%).

Labels do not consistently contain needed information. When foodborne illness was related to consumption of unpasteurized apple juice, consumers were not able to determine from the label which products were pasteurized. Many major manufacturers do not indicate whether their fresh juice product is pasteurized.

Consumers are not advised about potential risks for special populations. Raw milk sold in California must contain a warning statement, but other states may not have this requirement. Because of inconsistencies in labeling, unpasteurized juice products may be given to infants. Also, products that contain honey do not include a warning about potential risk for infant botulism.

### Processing Technology

Consumers do not realize that pathogens can survive minimal processing, as evidenced by a recent *Escherichia coli* outbreak associated with fresh apple juice, which demonstrated that processors also may not recognize potential risks. A fresh apple juice manufacturer in northern California claimed its product was safe because the juice was squeezed in small batches and frozen immediately.

Freezing is not effective against *E. coli* O157:H7, but other methods are protective. Several methods have been developed to reduce pathogens and increase the safety of foods. Once these methods are verified as effective and safe, the food industry should be free to use them, and consumers should have the opportunity to select safer foods. In some cases, the regulatory approval process appears to hinder rather than facilitate the safer handling of food.

Food irradiation, exposing food to high levels of electromagnetic energy for specific purposes, has been approved for selected uses. A petition before the Food and Drug Administration to permit irradiation of meat and other muscle foods appears to have satisfied safety concerns, but approval has not yet been granted. The requirement to seek approval for each application of irradiation prevents rapid response in cases of foodborne outbreaks. Although this regulatory procedure may have been reasonable when irradiation was first introduced, it warrants a fresh look in view of the wealth of data now available on the safety and wholesomeness of food irradiation (16).

Attitude surveys and marketing experience consistently demonstrate that consumers will purchase irradiated food (17). National surveys indicate consumer concern about irradiation was lower than other food-related concerns. When specifically asked what they considered a serious health hazard, 29% identified irradiation, 77% identified bacteria, and 66% identified pesticides (1). The percentage of consumers concerned about irradiation has decreased significantly over time. In the late 1980s, 42% to 43% classified irradiation as a serious concern, decreasing to 29% in 1996. A relative ranking of food processing methods surveyed by the Gallup Organization found that irradiation, food preservatives, and chlorination generated similar concern (18).

In a nationwide Food Marketing Institute survey, 69% of consumers indicated they were very or somewhat likely to purchase products irradiated to kill bacteria or other microorganisms (1). Surveys completed in several areas of the country indicate 60% to 70% of consumers would prefer irradiated food (17). In one study, information about irradiation increased interest in purchasing to 90%, and education plus food samples increased purchase intent to 99%.

Consumers have purchased irradiated food in select locations across the United States since 1992 (17). Fruits from the Mainland and Hawaii have sold well in the Midwest and California (L. Wong, pers. comm.). Irradiated chicken gained over 60% of market share when priced 10% lower than nonirradiated chicken and 47% when priced the same (J.A. Fox, pers. comm.).

Irradiated food is not widely available. Special interest groups threaten companies that exchange information about irradiation processing. Consumers, however, appear to prefer irradiated foods when the benefits of these foods are endorsed by health professionals. Food manufacturers and retailers should offer consumers the choice of safety-enhanced, energy-pasteurized irradiated food.

### Communicating with the Public

To respond to consumers' need for information, a multifaceted program is needed. The HACCP strategy, which teaches consumers to critically think through the food safety process to determine how foodborne illness could occur, has been effective (19,20). The HACCP approach to home food preservation is logical and highlights key control points (21).

Consumers should be informed that microorganisms are ubiquitous in the environment, found on raw products of animal or plant origin. Pathogens may survive minimal processing and preservation treatments. People may introduce pathogens during any stage of food processing or handling, including just prior to consumption. Foodborne illness can range from mild to severe and life-threatening with chronic complications. People have control and can reduce risks.

Communicating food safety information to the public effectively is another challenge. Consumers obtain most of their information on food, nutrition, and science from the media; television is cited most frequently, and newspapers and magazines follow (22,23). Brochures enforce messages and serve as useful references, although they are not as widely seen as media stories.

Developing messages with the press should be a primary activity of a food safety education program. Consumers judge a message by the credibility of the person conveying it, its appeal to their common sense, and the frequency of the message (24). Media presentations can motivate people to listen and change behavior. Consultants from the USDA hotline say, "We've seen an explosion in media coverage of food safety, and callers want more detailed explanations of things they read and hear" (25).

Information on safe food handling must be motivating and memorable. Stories that capture the public's attention are personal. They relate life experiences of people with whom the public can identify. Stories of the consequences of mistakes are memorable. They can be touching, humorous, or grotesque. It is easy to visualize and remember the infected bakery worker who made 5,000 people ill when he mixed a vat of buttercream frosting with his bare hands and arms despite bouts with diarrhea (26).

Stories can be heartrending, as in the experiences of a family who lost a child to *E. coli* O157:H7 infection. It is difficult to document the effectiveness of vivid accounts of doing things right or wrong. However, when Washington state carried extensive coverage linking the outbreak to undercooked hamburgers, 13% of the men said they ate undercooked hamburger, compared with 38% in Colorado (25).

### Conclusions

Consumer concerns about foodborne illness can motivate change in regulatory and industry use of technology, product labeling, and consumer education.

### New Technologies

The food industry has both a right and a responsibility to use safe and effective technology to enhance the safety of the food supply. Regulatory authorities should expediently evaluate and facilitate new technologies, such as food irradiation, laser light treatment, and high-pressure processing, which enhance food safety. Health professionals, the food industry, and regulators should challenge special interest groups that distort information and strive to limit consumer choice.

### Improved Labeling

Processed and packaged food should bear labels that clearly indicate how food should be handled. Labels should include warnings about special risks to select populations. Benefits from special processing that can reduce microbes should also be encouraged.

### Consumer Education

Consumers need to appreciate the seriousness of foodborne diseases. They must learn to recognize unsafe food-handling practices, the latency period for some microbes, and the symptoms of foodborne disease. They need to understand how to protect themselves through kitchen and personal hygiene, including thoroughness and frequency of hand washing, temperature control, and safe food choices such as foods processed by heat or energy pasteurization. Young people should be reached through age-specific school curricula, such as personal hygiene and special "living skills" units that address food safety and diet. Food industry and health educators should work with the media to develop interesting and timely messages to increase consumer knowledge about safe food handling. Messages must be consistent, science-based, frequent, and personalized.

### References

1. Abt Associates Inc. Trends in the United States, consumer attitude and the supermarket 1996. Washington (DC): Food Marketing Institute; 1996.
2. Institute of Food Technologists' Expert Panel on Food Safety and Nutrition. Scientific status summary, foodborne illness: role of home food-handling practices. Food Technology 1995;49:119-31.
3. Williamson DM, Gravani RB, Lawless HT. Correlating food safety knowledge with home food-preparation practices. Food Technology 1992;46:94-100.
4. Bryan F. Risks associated with vehicles of foodborne pathogens and processes that lead to outbreaks of foodborne diseases. Journal of Food Protection 1988;51:663-73.
5. Bean NH, Griffin PM. Foodborne disease outbreaks in the United States, 1973-1987; pathogens, vehicles, and trends. Journal of Food Protection 1990;53:804-17.
6. Dumagan JC, Hackett JW. Almost half of the food budget is spent eating out. Food Review 1995;18:37.
7. Martin R. Foodborne disease threatens industry. National Restaurant News 1990;24:27,30.
8. Doyle MP. Food safety in the 21st century. Dairy Food Environ Sanit 1993;13:383.
9. Food Institute. Population trends 1996-2050. Fair Lawn (NJ): The Institute; 1996. p. 31.
10. Fein SB, Jordan CT, Levy AS. Foodborne illness: perceptions, experience, and preventive behaviors in the United States. Journal of Food Protection 1995;58:1405-11.
11. Tauxe RV. Epidemiology of *Campylobacter jejuni* infections in the United States and other industrialized nations. In: Nachamkin J, Blaser MJ, Tompkins LS, editors. *Campylobacter jejuni*: current status and future trends. Washington (DC): American Society for Microbiology; 1992. p. 9-19.
12. Ollinger-Snyder P, Matthews ME. Food safety issues: press reports heighten consumer awareness of microbiological safety. Dairy, Food and Environmental Sanitation 1994;14:580-9.
13. Schafer RB, Schafer E, Bultena GL, Hoiberg EO. Food safety: an application of the health belief model. Journal of Nutritional Education 1993;25:17-24.
14. Food Marketing Institute. Opinion research, trends 90, consumer attitudes & and the supermarket 1990. Washington (DC): The Institute; 1990.
15. Rhodes ME. Educating professionals and consumers about extended-shelf-life refrigerated foods. Food Technology 1991;45:162-4.
16. Diehl JF. Safety of irradiated foods. New York: Marcel Dekker, Inc.; 1995.
17. Bruhn CM. Consumer attitudes and market response to irradiated food. Journal of Food Protection 1995;58:175-81.
18. The Gallup Organization. Irradiation: consumers' attitudes. Princeton (NJ): Prepared for the American Meat Institute; 1993.
19. Hoban T. Consumer awareness and acceptance of bovine somatotropin. Survey conducted for the Grocery Manufacturers of America; 1994.
20. Hoban T, Kendall PA. Consumer attitudes about the use of biotechnology in agriculture and food production. Raleigh (NC): North Carolina State University; 1992.
21. Bruhn CM, Peterson S, Phillips P, Sakovich N. Consumer response to information on integrated pest management. Journal of Food Safety 1992;12:315-26.
22. United States Department of Agriculture. The Food Safety Educator 1996;1:3.
23. Beware the Norwalk virus. Food Talk (Winter) 1989;4.
24. Reicks J, Bosch A, Herman M, Krinke UB. Effectiveness of a food safety teaching strategy promoting critical thinking. Journal of Nutritional Education 1994;26:97-100.
25. Medeiros LC, George RT, Gruns K, Chandler C, Crusey S, Fittro J, et al. The safe food handling for occasional quantity cooks curriculum. Journal of Nutritional Education 1996;28:39-42.
26. United States Department of Agriculture, Food Safety and Inspection Service. A margin of safety: the HACCP approach to food safety education. Washington (DC): United States Department of Agriculture Information and Legislative Affairs; 1990.

